# Transcriptome of Tumor-Infiltrating T Cells in Colorectal Cancer Patients Uncovered a Unique Gene Signature in CD4^+^ T Cells Associated with Poor Disease-Specific Survival

**DOI:** 10.3390/vaccines9040334

**Published:** 2021-04-01

**Authors:** Salman M. Toor, Varun Sasidharan Nair, Reem Saleh, Rowaida Z. Taha, Khaled Murshed, Mahmood Al-Dhaheri, Mahwish Khawar, Ayman A. Ahmed, Mohamed A. Kurer, Mohamed Abu Nada, Eyad Elkord

**Affiliations:** 1 Cancer Research Center, Qatar Biomedical Research Institute (QBRI), Hamad Bin Khalifa University (HBKU), Qatar Foundation (QF), P.O. Box 34110 Doha, Qatar; mstoor@hbku.edu.qa (S.M.T.); vsnair@hbku.edu.qa (V.S.N.); rsaleh@hbku.edu.qa (R.S.); rotaha@hbku.edu.qa (R.Z.T.); 2 Department of Pathology, Hamad Medical Corporation, P.O. Box 3050 Doha, Qatar; kmurshed@hamad.qa; 3 Department of Surgery, Hamad Medical Corporation, P.O. Box 3050 Doha, Qatar; maldhaheri@hamad.qa (M.A.-D.); mkhawar@hamad.qa (M.K.); aahmed40@hamad.qa (A.A.A.); mkurer@hamad.qa (M.A.K.); mabunada@hamad.qa (M.A.N.); 4 Biomedical Research Center, School of Science, Engineering and Environment, University of Salford, Manchester M5 4WT, UK

**Keywords:** colorectal cancer, tumor microenvironment, T cells, transcriptomics

## Abstract

Colorectal cancer (CRC) is influenced by infiltration of immune cell populations in the tumor microenvironment. While elevated levels of cytotoxic T cells are associated with improved prognosis, limited studies have reported associations between CD4^+^ T cells and disease outcomes. We recently performed transcriptomic profiling and comparative analyses of sorted CD4^+^ and CD8^+^ tumor-infiltrating lymphocytes (TILs) from bulk tumors of CRC patients with varying disease stages. In this study, we compared the transcriptomes of CD4^+^ with CD8^+^ TILs. Functional annotation pathway analyses revealed the downregulation of inflammatory response-related genes, while T cell activation and angiogenesis-related genes were upregulated in CD4^+^ TILs. The top 200 deregulated genes in CD4^+^ TILs were aligned with the cancer genome atlas (TCGA) CRC dataset to identify a unique gene signature associated with poor prognosis. Moreover, 69 upregulated and 20 downregulated genes showed similar trends of up/downregulation in the TCGA dataset and were used to calculate “poor prognosis score” (ppScore), which was significantly associated with disease-specific survival. High ppScore patients showed lower expression of Treg-, Th1-, and Th17-related genes, and higher expression of Th2-related genes. Our data highlight the significance of T cells within the TME and identify a unique candidate prognostic gene signature for CD4^+^ TILs in CRC patients.

## 1. Introduction

Global cancer statistics have ranked colorectal cancer (CRC) as the fourth most commonly diagnosed cancer and associated it with high mortality in both sexes combined (incidence; 6.1%, mortality; 9.2% of total cancer cases) [1]. The advent of novel therapeutic approaches, primarily in the form of immunotherapy, and improved screening technologies led to steady decrease in mortality rates of common cancers over the past few decades. However, these declines have slowed down or even halted for some cancers in recent years [2]. Therefore, there is a dire need to explore novel targets and biomarkers for cancer progression.

Besides the molecular basis of CRC development, which results from alterations in genes that drive tumor formation, the in situ immune cell infiltrates in the tumor microenvironment (TME) bear immense significance in disease prognosis. Tumor “immune editing” has been implicated in disease development and progression [3], and the tumor-immune microenvironment (TIME) is widely recognized as responsible for dictating tumor progression and response to therapy [4]. Effective destruction of cancer cells by tumor-reactive T cells requires overcoming various immunosuppressive factors/mechanisms. Cell-mediated immunosuppressive factors in the TME are primarily released by T regulatory cells (Tregs) and myeloid-derived suppressor cells (MDSCs), both previously reported to be expanded in the colorectal TME [5,6]. However, the relationship between Tregs and tumor progression in CRC is less clear; some studies suggested that high infiltration of FoxP3^+^ Tregs is associated with a favorable prognosis in CRC [7,8], while others reported the association of Tregs with a poor prognosis and attributed it to Treg heterogeneity [9,10]. Moreover, while elevated levels of tumor-infiltrating cytotoxic T cells (CTLs) are associated with improved prognosis and outcomes of CRC [11], limited studies have reported a significant association between CD4^+^ T cell infiltration and survival; possibly attributing it to the different subsets and heterogeneity of CD4^+^ T cell populations [12]. In addition, “immunoscore” has been proposed as an additional tool for CRC prognosis [13]. Galon et al., investigated CD3^+^ T cells and CD8^+^ CTLs in the center of a tumor and at the invasive margin, and reported the immunological data as a more robust survival predictor than the histopathological methods in clinical practice to segregate between CRC stages [14].

Molecular and cellular profiling of immune cell populations, specifically T cells, bear great significance in understanding CRC development and progression. We have recently performed transcriptomic profiling of sorted CD4^+^ [15] and CD8^+^ [16] tumor-infiltrating lymphocytes (TILs) from bulk tumors of CRC patients with varying disease stages. We reported downregulation of Th1-mediated immune response and cytotoxicity-mediated genes but upregulation of epigenetic silencing-related genes in CD4^+^ TILs from CRC patients with advanced stage disease [15]. Moreover, epigenetic regulation-related genes and response to hypoxia were upregulated, while T cell/cell proliferation- and cell cycle-related genes were downregulated in CD8^+^ TILs in advanced stage CRC patients [16]. These findings highlighted the significance of CD4^+^ and CD8^+^ TILs in CRC patients during disease progression. In this study, we reanalyzed our data and performed comprehensive comparative analyses of transcriptomes of CD4^+^ with CD8^+^ TILs from CRC patients (irrespective of disease staging). Our data uncovered various genes and their associated pathways, which are differentially expressed in CD4^+^ and CD8^+^ TILs from CRC patients. Importantly, we found that genes associated with T cell co-stimulation were upregulated, while inflammatory response-related genes were downregulated in CD4^+^ TILs, compared with CD8^+^ TILs, among other genes/pathways. The clinical significance of our results was exploited via alignment with the cancer genome atlas (TCGA) for colon and rectal cancer (COADREAD) RNA-Seq dataset, which enabled us to identify a unique gene signature of CD4^+^ TILs associated with decreased disease-specific survival (DSS) in data from 512 CRC patients. We referred to this gene signature as a “poor prognostic” (pp) gene signature and scored our patient cohort based on its expression. We found that patients with a high ppScore showed lower CD4^+^ TILs than those with a low ppScore. Moreover, comparative analyses of their transcriptomes revealed impaired immune responses and downregulation of important pathways, such as DNA repair, in high ppScore patients. Thus, our data show the potential candidacy of a unique gene signature associated with poor DSS in CRC patients.

## 2. Materials and Methods

### 2.1. Study Design

Tumor tissue specimens from 18 CRC patients were used to sort pure CD4^+^ and CD8^+^ TILs. Libraries were generated from sorted cells for performing RNA-Seq. Various bioinformatics tools were utilized for analyses and visualization of RNA-Seq data. The raw data used in this study were generated previously in the same patient cohort [15,16] but were analyzed herein to uncover novel information. The study design has been clearly presented in [16] and summarized below.

### 2.2. Sample Collection and Storage

Tumor tissue (TT) specimens were collected from 18 treatment-naïve CRC patients, who undertook surgical tumor resection at Hamad Medical Corporation, Doha, Qatar. The clinical and pathological features of all participating patients are listed in Table 1. Written informed consents were collected from patients prior to sample collection. This study received ethical approvals from the Institutional Review Boards at Hamad Medical Corporation, Doha, Qatar (protocol no. MRC-02-18-012) and the Qatar Biomedical Research Institute, Doha, Qatar (protocol no. 2017-006).

TT specimens were cut into smaller sections and stored in freezing medium [40% RPMI-1640 medium (Life Technologies, New York, NY, USA), 50% fetal calf serum (FCS; Hyclone, GE Healthcare Life Sciences, Logan, UT, USA), and 10% dimethylsulphoxide (DMSO; Sigma-Aldrich, St. Louis, MO, USA)] to be used in batches for succeeding investigations, as previously described [16,17].

### 2.3. Dissociation of Tissues

Bulk TT specimens were mechanically dissociated to prepare cell suspensions, as previously described [16,17]. Briefly, frozen TT specimens were washed with phosphate-buffered saline (PBS) and a surgical scalpel was used to cut TT into small pieces (~2–4 mm). A GentleMACS dissociator (Miltenyi Biotec, Bergisch Gladbach, Germany) was used to perform cell dissociation without utilizing proteolytic enzymes. Resulting cell suspension was passed through a cell strainer (100 µM) to remove debris and cell aggregates, and washed multiple times with PBS prior to cell sorting.

### 2.4. Cell Sorting

Single cell suspensions from TT specimens were resuspended in 100 µL of flow cytometry staining buffer (PBS with 1% FCS and 0.1% sodium azide). An FcR Blocking Reagent (Miltenyi Biotec) was used to block Fc receptors (FcR) and a 7-AAD viability dye (eBioscience, San Diego, CA, USA) was used to gate live cells. Cells were stained with surface antibodies against CD3-allophycocyanin-Cy7 (clone SK7, BD Pharmingen, San Jose, CA, USA), CD4-phycoerythrin (clone RPA-T4, BD Pharmingen), and CD8-fluorescein isothiocyanate (clone RPA-T8; BD Pharmingen). Cells were washed twice with flow cytometry staining buffer prior to re-suspension in Pre-Sort buffer (BD Biosciences, Oxford, UK). A BD FACSAria III SORP cell sorter on BD FACSDiva software (BD Biosciences) was used for sorting pure CD4^+^ (7AAD^−^CD3^+^CD4^+^CD8^−^), and CD8^+^ (7AAD^−^CD3^+^CD4^−^CD8^+^) TILs. Relevant procedures were followed to ensure minimum sorter-induced cell stress (SICS), as previously described [18]. Flow cytometric analyses were performed on FlowJo V10 software (FlowJo, Ashland, OH, USA).

### 2.5. RNA Isolation and Amplification

RNA was isolated from sorted, pure CD4+ and CD8+ TILs from 18 CRC patients using an RNA/DNA/protein purification Plus Micro Kit (Norgen Bioteck Corporation, Thorold, Ontario, Canada) by following manufacturer’s protocol. RNA was amplified using a 5X MessageAmp™ II aRNA Amplification Kit (Invitrogen, Carlsbad, CA, USA) by following the manufacturer’s protocol. Concentrations of RNA were determined using Qubit RNA HS or Broad Range Assay Kits (Invitrogen, Carlsbad, CA, USA).

### 2.6. Library Preparation

cDNA libraries were generated from amplified RNA using an Exome TruSeq Stranded mRNA Library Prep Kit (illumina, San Diego, CA, USA) by following the manufacturer’s protocol, and as previously described [19]. Quality-passed libraries were processed for clustering using a TruSeq PE Cluster Kit v3-cBot-HS (illumina). The clustered samples were sequenced on an illumina HiSeq 4000 platform using a HiSeq 3000/4000 SBS kit (illumina).

### 2.7. RNA-Sequencing Data Processing and Analyses

RNA-Seq data were analyzed and illustrated using multiple bioinformatics tools. Pair end reads were quality-trimmed and aligned to the hg19 human reference genome in CLC Genomics Workbench 12 (Qiagen), as previously described [19]. The expression levels of transcripts were measured as TPM (Transcripts Per Million) mapped reads. Abundance data were successively subjected to differential gene expression analyses. The Z-scores were calculated from TPM values, as previously described [20], and used to generate heatmaps.

Volcano plots were generated using OrignPro 2020 software (OriginLab Corporation, Northampton, MA, USA) with log2 FC > 2 and *p* value cutoff < 0.05. The Gene Ontology (GO) analysis and Kyoto Encyclopedia of Genes and Genomes (KEGG) pathway enrichment analyses of differentially expressed genes (DEGs) were performed by the Database for Annotation, Visualization and Integrated Discovery (DAVID) tool, as previously described [21].

### 2.8. Data Alignment with the Cancer Genome Atlas (TCGA) Colorectal Cancer

The top 100 upregulated genes and 100 downregulated genes from CD4^+^ and CD8^+^ TILs comparison was selected for analysis in a TCGA CRC dataset. Upregulated and downregulated genes aligned with TCGA data were used to identify the “poor prognosis gene signature score”. The ppScore was calculated as the ratio of the average expression of the aligned upregulated genes to the average of the aligned downregulated genes. The percentile calculations for the ppScore were performed to determine CRC patients with a high ppScore and a low ppScore. Therefore, the poor prognosis gene signature was identified from data of our cohort and its prognostic significance was confirmed by TCGA datasets (512 CRC patients). The survival and phenotype data were obtained from TCGA database to plot Kaplan–Meier survival curve and calculate log-rank *P* values using Graphpad Prism 8. Multivariate analyses/Cox proportional-hazard model were performed using EZR (Easy R) statistical software. Chi-squared χ2 test was used to determine the statistical significance of the distribution of CRC patients across different disease stages.

### 2.9. TCGA Immune Estimations

Gene expression of 69 upregulated and 20 downregulated genes, which are included in the gene signature, were downloaded from the UCSC Xena platform (http://xena.ucsc.edu/) for 512 CRC (COAD) patient cohort. The immune infiltration data (CD4^+^ and CD8^+^ TILs) for the aforementioned patients were obtained from CIBERSORT immune fractions (https://gdc.cancer.gov/). Correlation analyses between the gene expression and CD4/CD8 TILs fraction were performed using a regression-based method. The obtained correlation coefficients were plotted as heatmaps. The *p* value < 0.05 is considered as statistically significant.

## 3. Results

### 3.1. Comparing Transcriptomes of CD4^+^ and CD8^+^ Tumor-Infiltrating Lymphocytes in Colorectal Cancer Patients

**Figure 1:**
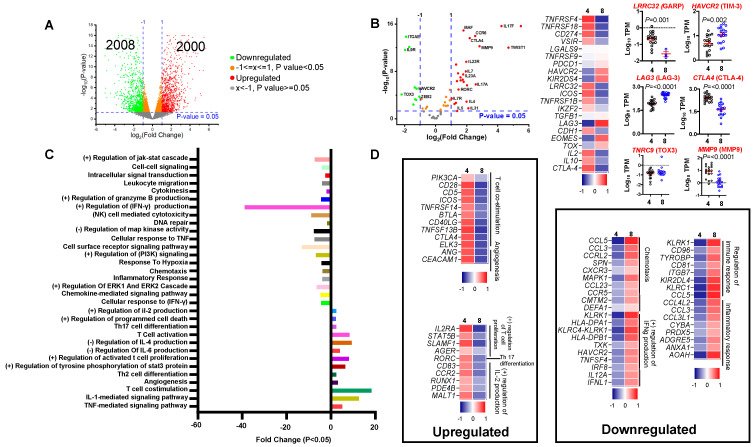
Differential gene expression analyses of CD4^+^ and CD8^+^ TILs. Transcriptomic profiling and differential gene expression analyses were performed from FACS sorted pure CD4^+^ and CD8^+^ TILs from 18 CRC patients. Volcano plot shows the significantly upregulated (red; 2000 genes) and downregulated (green; 2008 genes) with *p* value < 0.05 and FC > 1 in CD4^+^ TILs, compared with CD8^+^ TILs (**A**). Volcano plot shows the deregulation of selected genes in CD4^+^ vs. CD8^+^ TILs and representative heatmap of Z-score for significantly downregulated or upregulated genes in CD4^+^ TILs and CD8^+^ TILs. Scatter plots show the Log10 TPM of selected genes ± SEM in CD4^+^ TILs and CD8^+^ TILs (**B**). Functional annotations of significantly downregulated and upregulated genes (with *p* < 0.05) were analyzed by DAVID web-based tool. Bars show the fold enrichment of pathways that were significantly downregulated or upregulated in CD4^+^ TILs from CD4^+^ TILs vs. CD8^+^ TILs (**C**). Heatmaps for selected upregulated and downregulated pathways and show the Z-score for genes that were differentially expressed in CD4^+^ TILs vs. CD8^+^ TILs (**D**).

The presence of TILs in CRC patients has been associated with improved clinical outcomes [22,23]. However, heterogeneity of TILs contributes to both pro- and antitumor roles [24]. To understand the differences between roles of T cells in the colorectal TME, we performed differential gene expression analyses for transcriptomes of sorted, pure CD4^+^ and CD8^+^ TILs. We used CD8^+^ TILs as controls due to their widely accepted antitumor roles [25] and compared with the transcriptome of CD4^+^ TILs due to their dual roles in tumor progression. We found 4008 differentially expressed genes (DEGs) between CD4^+^ and CD8^+^ TILs (log2 FC ≥ 2 and *p* value cutoff ≤ 0.05). A total of 2000 genes were upregulated, while 2008 genes were downregulated in CD4^+^ TILs (Figure 1A). Of note, genes known to be expressed in CD4^+^ or CD8^+^ T cells were utilized as controls to confirm the purity of the analyzed T cell subsets.

### 3.2. Differential Expression of T Cell Related Genes

We then investigated DEGs between CD4^+^ and CD8^+^ TILs, focusing on T cell related genes (Figure 1B, Supplementary Materials Table S1). We found that *IL4*, *IL5*, *IL17*, *CCR6*, and *RORC* were upregulated, while *ZEB2*, *IL91R* and *ITGAE* were downregulated in CD4^+^ TILs. Importantly, immune checkpoint genes, including *CTLA4* and *ICOS*, were upregulated, while *HAVCR2* (gene encodes TIM-3) and *LAG3* were downregulated in CD4^+^ TILs, with no significant changes recorded in *PDCD1* (gene encodes PD-1) expression between CD4^+^ and CD8^+^ TILs. Moreover, we found that *TOX* gene, representing a member of the thymocyte selection-associated high mobility group box protein (TOX) family implicated in T cell exhaustion [26], was downregulated in CD4^+^ TILs, compared to CD8^+^ TILs (Figure 1B). However, there was no significant difference in the expression of TOX3 *(TNRC9)* between CD4^+^ TILs and CD8^+^ TILs (Figure 1B). In order to validate the association between gene signature and protein expression on TILs, we investigated the expression levels of selected dysregulated genes on CD4^+^ and CD8^+^ TILs from six patients out of our patient cohort (Supplementary Materials Figure S1). These genes include immune checkpoints; *HAVCR2* (gene for TIM-3), *ICOS* and *LAG3*. In agreement with the transcriptomic data, we found that TIM-3 protein was expressed at higher levels on CD8^+^ TILs compared to CD4^+^ TILs (*p* = 0.035, Supplementary Materials Figure S1A). LAG-3 protein levels were also higher on CD8^+^ TILs compared to CD4^+^ TILs, but did not show statistical significance (Supplementary Materials Figure S1B). In contrast, ICOS protein was expressed at higher levels on CD4^+^ TILs (*p* = 0.005, Supplementary Materials Figure S1C).

### 3.3. Functional Pathways Enriched in CD4^+^ TILs Compared to CD8^+^ TILs

To identify the functional pathways of DEGs from CD4^+^ vs. CD8^+^ TILs comparison, we performed gene ontology (BP) and KEGG pathway analyses using the DAVID platform. We found that genes involved in the regulation of IFNγ/granzyme B production, IL-2 production, response to hypoxia, chemotaxis, and inflammatory response were downregulated in CD4^+^ compared to CD8^+^ TILs (Figure 1C,D). In contrast, T cell co-stimulation-, activation-, proliferation-, Th17 differentiation-, IL-4/IL-6 production- and angiogenesis-related genes were upregulated in CD4^+^ compared to CD8^+^ TILs (Figure 1C,D). The upregulated genes include *ICOS, BTLA, ANG, IL2RA, RORC, CD83*, and *MALT1* (Figure 1D), while downregulated genes include *CCL3, CCL5, HAVCR2, TNFSF4, IFNL1, CD81*, and *PRDX5* (Figure 1D).

### 3.4. Aligning Gene Profile of CD4^+^ TILs from CRC Patients with TCGA Revealed a Distinct Gene Signature Associated with Poor Disease-Specific Survival

**Figure 2:**
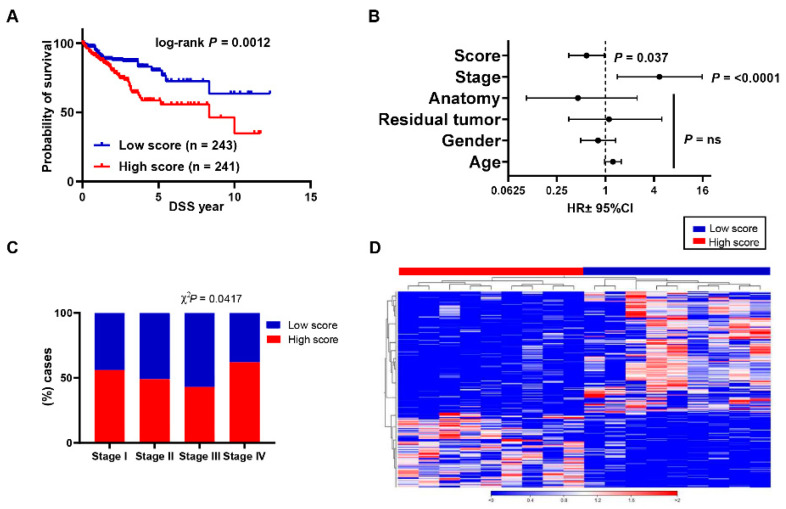
Evaluation of ppScore in TGCA COADREAD dataset. Kaplan–Meier curves for disease-specific survival were compared between patients with high (top 50%) and low (bottom 50%) ppScores. The number (*n*) of patients in each of the ppScore groups and the log-rank *p* value from Mantel–Cox test are indicated (**A**). Multivariate analyses using Cox proportional-hazard model evaluating the prognostic indication for the ppScore, disease stage (Stages I, II, III and IV), anatomic locations (7 different locations), residual disease (yes, no), gender (male, female) and age (<55, 55–64, 65–74, >74 years of age), and for disease-specific survival (**B**). Data shown is the hazard ratio (HR) ± 95% confidence interval (CI) and the multivariate *p* values are indicated. Distribution of patients with a high and low ppScore across different stages using Chi-squared (χ2) test (Stages I, II, III and IV) (**C**). Our patient cohort was classified into two groups: high ppScore and low ppScore, and differential analyses were performed. Hierarchical clustering shows high ppScore and low ppScore (9 patients each) on differentially expressed transcripts in CD4^+^ TILs. Expression level is depicted as a color code and each column represents a sample and each row represents a transcript (**D**).

The top 100 upregulated and downregulated genes from CD4^+^ TILs vs. CD8^+^ TILs were annotated on TCGA COADREAD RNA-Seq dataset to identify ppScore. Of these 100 upregulated genes, 98 genes were annotated in TCGA CRC RNA-Seq dataset, of which 69 genes (70.4%) had higher expression in patients with poorer DSS. For the 100 downregulated genes, 96 genes were annotated in TCGA dataset, of which 20 genes (20.8%) had lower expression in patients with poorer DSS. The selected 69 upregulated and 20 downregulated genes were used as the “poorer prognosis gene signature score” (Supplementary Materials Table S2). The ppScore was calculated as the ratio of the average expression of the 69 upregulated genes to the average of the 20 downregulated genes. The percentile calculations for the ppScore were performed to determine CRC patients with a high ppScore (top 50%) or a low ppScore (bottom 50%). Patients in TCGA dataset were classified into two groups, either with a low (*n* = 241) or a high ppScore (*n* = 243). Interestingly, we found that patients with a high ppScore had significantly poorer DSS, compared to those with a low ppScore (log-rank *p* = 0.0012, Figure 2A). Moreover, multivariate analyses using Cox proportional-hazard model showed that our identified ppScore was an independent prognostic factor for DSS, even in the presence of disease stage as an indicator (*p* = 0.037, HR ± 95%, Figure 2B). Patients with a high ppScore were more likely to have advanced disease stages (χ2 *P* = 0.042, Figure 2C). Taken together, the TCGA analyses support that the identified ppScore could predict DSS in CRC patients. Next, we classified our patient cohort into two groups based on the percentile cutoff of the threshold value; high ppScore (*n* = 9) and low ppScore (*n* = 9) patients (Supplementary Materials Table S3). Gene expression analyses showed that 958 genes were differentially expressed in high ppScore patients with FDR cutoff < 0.01, of which 441 were upregulated and 517 were downregulated (Figure 2D).

**Figure 3:**
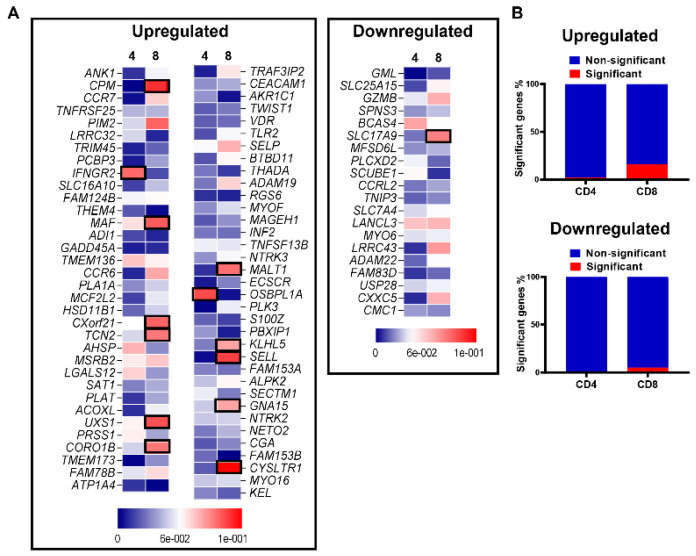
T cell infiltration in high and low ppScore CRC patients. CD4^+^/CD8^+^ TILs’ distribution data were deconvoluted from TCGA datasets of 512 CRC patients. Correlation analyses were then performed on the identified gene signature from our data and T cell infiltration data from TCGA, using a regression-based method. The correlation coefficient R values were plotted as heatmaps, where the significantly correlated genes (*p* < 0.05) were marked in black boxes (**A**). The bar plots show the percentages of significant genes from downregulated and upregulated gene signature panel in CD4^+^ and CD8^+^ TILs (**B**).

Next, we correlated the expression of genes included in the signature cluster with CD4^+^ and CD8^+^ infiltrated from the deconvoluted COAD-TCGA dataset. We have calculated the correlation coefficient, “R”, and their significance, “*p* value”, to identify a plausible correlation of gene expression with the CD4 and CD8 fraction. Interestingly, we found that the vast majority of genes showed very weak correlations in either T cell subset with statistically non-significant *p* values (Figure 3A,B). From the upregulated panel, only 2.8% and 15.9% genes showed a statistically significant correlation with CD4^+^ and CD8^+^ TILS, respectively (*p* < 0.05, Figure 3A,B). Furthermore, in the downregulated panel, none of the genes from CD4^+^ TILs, while only 5% from CD8^+^ TILs, showed statistical significance (*p* < 0.05, Figure 3A,B). Notably, none of the genes showed strong positive or negative correlation coefficient with gene expression. Altogether, these data confirm that the identified gene signature is an independent factor and has no potential correlation with CD4^+^ or CD8^+^ TILs fraction.

### 3.5. Differences in Tumor-Infiltrating T Cells between CRC Patients with High and Low ppScore

**Figure 4:**
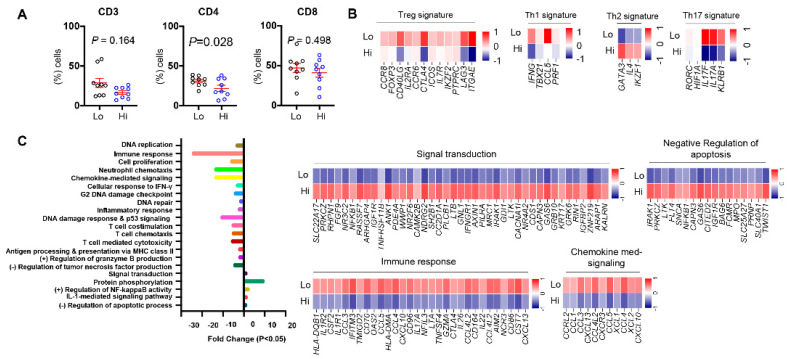
Characterization of high ppScore and low ppScore CRC patients. The 18 patients’ cohort was classified into two groups; high ppScore and low ppScore, as mentioned before. Scatter plots show the percentage of CD3^+^, CD4^+^ and CD8^+^ TILs ± SEM in high and low ppScore patients (**A**). Heatmaps show the Z-score of Treg- Th1-, Th2- and Th17- related genes in CD4^+^ TILs from high and low ppScore patients (**B**). Functional annotations of significantly downregulated and upregulated genes (with *p* < 0.05) were analyzed by a DAVID web-based tool. Bars show the fold enrichment of pathways that were significantly downregulated or upregulated in CD4^+^ TILs from high ppScore vs. low ppScore patients. Heatmaps for selected upregulated and downregulated pathways show the Z-score for genes that were differentially expressed in CD4^+^ TILs from high ppScore vs. low ppScore patients (**C**).

Next, we investigated the density of T cell infiltration in high and low ppScore patients in our cohort. We found that the levels of CD3^+^ TILs were less in patients with a high ppScore than a low ppScore, although it did not reach statistical significance, plausibly due to small sample size (Figure 4A). Interestingly, there was a significant reduction in levels of CD3^+^CD4^+^ TILs in patients with a high ppScore compared to a low ppScore. Moreover, there was no difference in the levels of CD3^+^CD8^+^ TILs between high and low ppScore patients (Figure 4A). These results prompted us to investigate the transcriptomes of CD4^+^ TILs from patients from the two patient groups to identify the potential T cell subtype(s). In this pursuit, we investigated differences in the expression levels of various Treg/Th1/Th2 and Th17 signature genes between patients with a high vs. a low ppScore (Figure 4B). Interestingly, we found that Treg signature genes, including *CCR8*, *FOXP3*, *CD40LG*, *IL2RA*, *CCR6*, *CTLA4*, *ICOS*, *IL7R*, *IKZF2*, *PTPRC*, *LAG3*, and *ITGAE*; Th1 signature genes, including *IFNG*, *TBX21*, *IRF8*, *CCL5*, and *PRF1*; and Th17 signature genes, including *RORC*, *HIFIA*, *IL17F*, *IL17A*, and *KLRB1* were significantly downregulated in CD4^+^ TILs in patients with a high ppScore compared with low ppScore patients. On the other hand, Th2 signature genes, including *GATA3*, *IL4* and *IKZF1*, were significantly upregulated in CD4^+^ TILs in patients with a high ppScore, compared with low ppScore patients (Figure 4B).

### 3.6. Differentially Expressed Genes and Associated Pathways between CRC Patients with High and Low ppScore

We then identified potential pathways, which were significantly deregulated in CD4^+^ TILs, from CRC patients with s high ppScore compared with low ppScore patients. Interestingly, we found that genes related to immune/inflammatory response, DNA replication/repair, T cell chemotaxis/stimulation, MHC class II presentation, response to IFNγ, and granzyme B production/T cell mediated cytotoxicity were all downregulated in CD4^+^ TILs from patients with a high ppScore compared to patients with low ppScores (Figure 4C). In contrast, protein phosphorylation-, NF-κB activity-, IL-1-mediated signaling- and negative regulation of apoptosis-related genes were upregulated in patients with high ppScores (Figure 4C). The upregulated genes in high ppScore patients included *IGF1R*, *IFNGR1*, *FLT4*, *PRNP* and *TWIST1*, among others (Figure 4C), while downregulated genes included *HLA-DQB1*, *CCL3*, *CCL4*, *IL22*, *CXCR3* and *CXCL10*, among others (Figure 4C). These results reflect the potential impaired activity of CD4^+^ TILs in patients with high ppScores and reiterate the significance of the identified gene signature, which affects pathways that could potentially contribute to poor prognosis via aberrant antitumor immune responses.

## 4. Discussion

Our data reiterate that CD4^+^ and CD8^+^ TILs comprise heterogenous populations, perform distinct roles, and affect unique pathways which dictate CRC tumor progression. However, the proper functionality of T cells is of paramount significance in antitumor immunity, and it has been shown that the activities of tumor-infiltrating T cells are differentially affected by the TME [27]. The functional impairment of T cells can be evident from the expression levels of immune checkpoints and T cell exhaustion markers [28]. We found that the majority of immune checkpoint genes were overexpressed in CD8^+^ TILs compared to CD4^+^ TILs, potentially suggesting T cell exhaustion and dysfunction of CTLs in the TME of CRC patients. Importantly, the downregulation of genes related to T cell activation/proliferation and co-stimulation in CD8^+^ TILs, compared to CD4^+^ TILs in our data, supported these findings. In addition, identification of such gene signatures associated with T cell dysfunction can also serve as predictive biomarkers for therapy. Jiang et al., showed that gene signatures related to T cell dysfunction and exclusion from the TME can predict a therapy response to immune checkpoint inhibitors, anti-PD-1 and anti-CTLA4 mAbs, in melanoma patients [29]. Gene signatures could be utilized as tools to distinguish potential cancer patients who could respond better to immunotherapies, including immune checkpoint inhibitors, based on the characterization of their tumor nature, “hot tumors”, i.e., T cell-inflamed tumors vs. “cold tumors”, i.e., T cell desert or noninflamed tumors in cancer patients [16,30].

The transcriptomes of CD4^+^ and CD8^+^ TILs also uncovered potential genes and their associated pathways, which reflect their roles in the TME. For instance, our data showed that *TGFβ1* gene encoding TGF-β, which is known to inhibit T effector cell functions, promote Treg survival/differentiation, and support tumor invasion and metastasis [31,32], was expressed at higher levels on CD8^+^ TILs compared to CD4^+^ TILs. It has been reported that the expression of *TGFβ1* on CD8^+^ TILs can modulate their antitumor functionality, while a pharmacological blockade of TGF-β can restore their cytotoxic function [33]. Moreover, two recent reports have suggested that TGF-β blockade can efficiently prevent CRC metastasis in tumor models and can also enhance the efficacy of PD-L1 blockade [34,35]. These findings implicate that the presence of additional immune subversive mechanisms in the TME, apart from TGF-β release from FoxP3^+^ Tregs, and even tumor cells [32,36]. In a similar fashion, we found that *CAECAM*, which is known to act as a proangiogenic factor supporting vascularization within the TME [37], was overexpressed in CD4^+^ TILs compared to CD8^+^ TILs. This latter finding implicates the potential involvement of CD4^+^ TILs, more likely to be CD4^+^ Tregs, in promoting tumor angiogenesis. In contrast, CD8^+^ TILs showed elevated levels of inflammatory chemokine genes, *CCL3* and *CCL5,* which are involved in the trafficking and recruitment of other immune cell populations to hinder tumor growth [38]. Additionally, genes encoding the IFN-γ receptor subunits *IFNGR1* and *IFNGR2* were downregulated, whereas *IFNG* gene expression, per se, was upregulated in CD8^+^ TILs. In the canonical IFN-γ signaling pathway, IFN-γ subunit binds to its receptors and activates the antitumor immune cascade through activation of Janus kinases, JAK1 and JAK2 [39]. In this context, the downregulation of IFN-γ receptors could potentially impair the cytotoxic function of CD8^+^ TILs and fail to induce appropriate antitumor responses in the CRC microenvironment. The above findings could rationalize the impact of the identified gene signature on the function of TILs, and suggest potential immune mechanisms and signaling pathways which could impair CD8^+^ TIL function within the CRC microenvironment. It has been reported that IFNG receptor signaling is dispensable for the expansion, contraction, and memory differentiation of CD8^+^ T cells [40]. Moreover, rare multiple cutaneous squamous cell carcinoma was reported in a patient with a deficiency of IFNGR2 expression [41]. Interestingly, we found that the downregulation of IFNGR1/R2 was associated with the upregulation of T cell exhaustion genes, including *PDCD1* and *TOX*, on CD8^+^ TILs. These data show that CD8^+^ T cells in the CRC TME might be exhausted/less functional, and lose their Th1-mediated tumor elimination competency. Additionally, we investigated the pattern of expression of methylation-related genes in patients with high and low ppScores to examine if there are any correlations between the expression of these epigenetic genes and the transcriptome signature of TILs. We found that CD4^+^ TILs in patients with high ppScores express low DNMT3B, KDM5B/6B and high HDAC5 and KAT2B levels, compared to those with low ppScores (data not shown). This is consistent with previous findings showing that the DNMT3B gene was downregulated in CD4^+^ TILs from CRC patients with advanced stages [15], indicating that transcriptional silencing through DNA methylation in CD4^+^ TILs could be associated with poor prognosis; KAT2B is important to skew Th1 differentiation into Tregs [42]; HDACs, including HDAC5, could prevent the induction and differentiation of Th1 cells [43,44], and reduced levels of KDM5B and KDM6B can positively skew Th2 differentiation [45,46]. However, IFN-γ and their receptor expression in CD8^+^ T cells may also be influenced by epigenetic modifications [47] and, therefore, functional investigations are necessitated to confirm impaired IFN-γ release by CD8^+^ TILs in CRC patients.

Aligning the top deregulated genes in CD4^+^ TILs with TCGA led us to identify a unique poor prognosis gene signature associated with reduced DSS in CRC patients. We found that this gene signature is an independent prognostic indicator, independent of demographic features of CRC patients. Importantly, this gene signature corresponded with clinical staging, as a high ppScore was mainly present in CRC patients with advanced stage disease. Moreover, we found that patients with high and low ppScores presented with a distinct gene profile, as evident from the hierarchical clustering of genes.

Next, we investigated the differences in T cell infiltration between CRC patients with low and high ppScores. Oure data revealed that patients with a high ppScore had significantly lower infiltration of CD4^+^ TILs. These results prompted us to investigate the different T cell subsets in the TME of patients with high and low ppScores. We found that genes related to Treg, Th1, and Th17 cells were highly expressed in patients with a low ppScore, while Th2-related genes were highly expressed in patients with a high ppScore. These data reflect that CRC patients with poor prognosis have less Treg, Th1 and Th17 infiltration, while patients with better prognosis have higher levels of these cells in the TME but reduced levels of Th2 cells. Th1 cells are crucial for the proliferation of CTLs, and Th1 cytokines inhibit the generation of Th2 [48]. Thus, lower Th1 cells in patients with a high ppScore may consequently lead to increased Th2 cells in these patients, as reported in this study. Moreover, as mentioned above, elevated FoxP3^+^ Treg levels in the TME have been associated with favorable prognosis in CRC patients [8]. In addition, Th1 levels have also been shown to be strongly associated with improved prognosis in CRC patients [49]. However, Th17 cell levels have been shown to be involved in pro- and antitumor roles [50], and evidences have been provided on their negative influence on disease prognosis in CRC patients [51]. The combined effects of these preferential accumulations of different T cell subsets lead to a reduction in immune cell response, T cell activation/chemotaxis, and other critical antitumor immune response-related pathways in patients with high ppScores, indicating a potential contribution to worsened disease outcomes in these patients. Moreover, we found that genes related to the negative regulation of apoptosis were upregulated in CD4^+^ TILs, compared with CD8^+^ TILs in patients with a high ppScore. This upregulation of antiapoptotic genes could lead to increase in levels of CD4^+^ TILs and shift the balance between Th cells and CTLs in the TME, which favors tumor progression [52]. It has been reported that targeted elimination of tumor cells by CTLs is mediated by the stimulation of apoptosis cascade through the perforin/granzyme pathway [53]. Here, we found that expression levels of *GRZMA* and *PRF1* genes were lower in patients with a high ppScore compared to patients with a low ppScore, potentially suggesting the suppression of CTLs in these patients and implicating that the identified gene signature could be utilized as a potential predictive prognostic biomarker in CRC patients.

Importantly, we found genes related to DNA replication, damage and repair were also downregulated in patients with high ppScore. These results bear significance due to the relevance of genomic instability and disease outcomes in CRC patients. CRC patients with hypermutated tumors exhibiting deficiency in the DNA mismatch–repair system and microsatellite instability respond well to immunotherapy [54]. Therefore, the poor prognostic gene signature identified in this study could have important potential clinical implications.

### 4.1. Limitations and Future Directions

In this study, we performed transcriptomic analyses of CD4^+^ and CD8^+^ TILs from a relatively small cohort of CRC patients. Additionally, limitations associated with RNA amplification and RNA-Seq analyses could lead to potential bias [55], which cannot be fully neglected, and confirmatory flow cytometric analyses would endorse these findings. Importantly, these data could be further strengthened by performing functional analyses on CD4^+^ TILs considering pathways, such as TNF-signaling and IL-1-mediated signaling/co-stimulation pathways, which were upregulated in these cells. Functional studies would also ascertain the impact of downregulated genes on the functional capacity of CD8^+^ TILs in eliciting potent antitumor immune responses. CD4^+^ T cell subsets, which are indeed responsible for prognostic differences, could also be explored further in a similar approach. In addition, detailed immune cell profiling using flow cytometry, coupled with single-cell RNA-Seq analyses from whole tumors, would assist in a comprehensive categorization of TILs present within the TME; this is particularly helpful in determining the level of tumor immunogenicity and predicting the host immune response to certain cancer immunotherapy. Moreover, quantifying different subsets of TILs and correlating the results with the upregulation of our identified gene signature would rule out the possible impact of CD4^+^ vs. CD8^+^ T cell numbers (CD4^+^:CD8^+^ ratio) in the TME. Lastly, in addition to TCGA datasets, the identified gene signature could be further validated in samples from larger cohorts of CRC patients and with varying disease stages, and RT-qPCR gene expression analysis could be employed to validate the identified gene signature in CRC tumor samples.

### 4.2. Conclusions

Our findings extend knowledge on the potential roles of CD4^+^ and CD8^+^ TILs in CRC patients. The differentially regulated genes and their associated pathways reflect how these cells could contribute to CRC progression. However, functional studies are required to ascertain the precise roles of these cells. We identified that a high Th2 gene signature and a low Treg/Th1/Th17 gene signature in patients with a high ppScore is associated with poor DSS. This identified gene signature takes into consideration multiple parameters in assessing disease prognosis since it is not linked to TNM staging, but it is based on the identification of dysregulated genes in TILs from CRC patients. Therefore, the ppScore can potentially be utilized as an additional prognostic indicator engulfed within precision medicine protocols. However, further validations should strengthen its clinical significance.

## Figures and Tables

**Table 1:** Clinical and pathological features of the study cohort.

	CRC Patients
**Number**	18
**Age** (median)	62 (23–78)
**Gender** (Male:Female)	10:8
**TNM stage**	
I	5
II	5
III	3
IV	5
**Tumor histological grade**	
G2 (Moderately differentiated)	17
G3 (Poorly differentiated)	1
**Lymphovascular invasion**	2
**MSI-H/BRAF mutation**	1 *
**Tumor location**	
Cecum	3
Ascending colon	2
Decending colon	3
Sigmoid	5
Rectosigmoid	5

* Possibility of Lynch syndrome.

## Data Availability

The data presented in this study are available on request from the corresponding author.

## Supplementary Materials

The following are available online at https://www.mdpi.com/article/10.3390/vaccines9040334/s1, Figure S1: Expression levels of selected dysregulated genes on CD4^+^ and CD8^+^ TILs, Table S1: T cell-related genes, Table S2: CD4^+^ TILs Poor Prognosis Score (ppScore) gene list, Table S3: Patient ppScores.

## References

[1] Bray F., Ferlay J., Soerjomataram I., Siegel R.L., Torre L.A., Jemal A. (2018). Global cancer statistics 2018: GLOBOCAN estimates of incidence and mortality worldwide for 36 cancers in 185 countries. CA Cancer J. Clin..

[2] Siegel R.L., Miller K.D., Jemal A. (2020). Cancer statistics, 2020. CA Cancer J. Clin..

[3] Chew V., Toh H.C., Abastado J.P. (2012). Immune microenvironment in tumor progression: Characteristics and challenges for therapy. J. Oncol..

[4] Whiteside T.L. (2008). The tumor microenvironment and its role in promoting tumor growth. Oncogene.

[5] Syed Khaja A.S., Toor S.M., El Salhat H., Ali B.R., Elkord E. (2017). Intratumoral FoxP3(+)Helios(+) Regulatory T Cells Upregulating Immunosuppressive Molecules Are Expanded in Human Colorectal Cancer. Front. Immunol..

[6] Toor S.M., Syed Khaja A.S., El Salhat H., Bekdache O., Kanbar J., Jaloudi M., Elkord E. (2016). Increased Levels of Circulating and Tumor-Infiltrating Granulocytic Myeloid Cells in Colorectal Cancer Patients. Front. Immunol..

[7] Hu G., Li Z., Wang S. (2017). Tumor-infiltrating FoxP3(+) Tregs predict favorable outcome in colorectal cancer patients: A meta-analysis. Oncotarget.

[8] Xu P., Fan W., Zhang Z., Wang J., Wang P., Li Y., Yu M. (2017). The Clinicopathological and Prognostic Implications of FoxP3(+) Regulatory T Cells in Patients with Colorectal Cancer: A Meta-Analysis. Front. Physiol..

[9] Lin Y.C., Mahalingam J., Chiang J.M., Su P.J., Chu Y.Y., Lai H.Y., Fang J.H., Huang C.T., Chiu C.T., Lin C.Y. (2013). Activated but not resting regulatory T cells accumulated in tumor microenvironment and correlated with tumor progression in patients with colorectal cancer. Int. J. Cancer.

[10] Saito T., Nishikawa H., Wada H., Nagano Y., Sugiyama D., Atarashi K., Maeda Y., Hamaguchi M., Ohkura N., Sato E. (2016). Two FOXP3(+)CD4(+) T cell subpopulations distinctly control the prognosis of colorectal cancers. Nat. Med..

[11] Reissfelder C., Stamova S., Gossmann C., Braun M., Bonertz A., Walliczek U., Grimm M., Rahbari N.N., Koch M., Saadati M. (2015). Tumor-specific cytotoxic T lymphocyte activity determines colorectal cancer patient prognosis. J. Clin. Investig..

[12] Kuwahara T., Hazama S., Suzuki N., Yoshida S., Tomochika S., Nakagami Y., Matsui H., Shindo Y., Kanekiyo S., Tokumitsu Y. (2019). Intratumoural-infiltrating CD4 + and FOXP3 + T cells as strong positive predictive markers for the prognosis of resectable colorectal cancer. Br. J. Cancer.

[13] Galon J., Pages F., Marincola F.M., Angell H.K., Thurin M., Lugli A., Zlobec I., Berger A., Bifulco C., Botti G. (2012). Cancer classification using the Immunoscore: A worldwide task force. J. Transl. Med..

[14] Galon J., Costes A., Sanchez-Cabo F., Kirilovsky A., Mlecnik B., Lagorce-Pages C., Tosolini M., Camus M., Berger A., Wind P. (2006). Type, density, and location of immune cells within human colorectal tumors predict clinical outcome. Science.

[15] Sasidharan Nair V., Saleh R., Taha R.Z., Toor S.M., Murshed K., Ahmed A.A., Kurer M.A., Abu Nada M., Al Ejeh F., Elkord E. (2020). Differential gene expression of tumor-infiltrating CD4(+) T cells in advanced versus early stage colorectal cancer and identification of a gene signature of poor prognosis. Oncoimmunology.

[16] Saleh R., Sasidharan Nair V., Toor S.M., Taha R.Z., Murshed K., Al-Dhaheri M., Khawar M., Petkar M.A., Abu Nada M., Al-Ejeh F. (2020). Differential gene expression of tumor-infiltrating CD8(+) T cells in advanced versus early-stage colorectal cancer and identification of a gene signature of poor prognosis. J. Immunother Cancer.

[17] Toor S.M., Murshed K., Al-Dhaheri M., Khawar M., Abu Nada M., Elkord E. (2019). Immune Checkpoints in Circulating and Tumor-Infiltrating CD4(+) T Cell Subsets in Colorectal Cancer Patients. Front. Immunol..

[18] Toor S.M., Sasidharan Nair V., Pfister G., Elkord E. (2019). Effect of pembrolizumab on CD4(+) CD25(+), CD4(+) LAP(+) and CD4(+) TIM-3(+) T cell subsets. Clin. Exp. Immunol..

[19] Vishnubalaji R., Sasidharan Nair V., Ouararhni K., Elkord E., Alajez N.M. (2019). Integrated Transcriptome and Pathway Analyses Revealed Multiple Activated Pathways in Breast Cancer. Front. Oncol..

[20] Malone B.M., Tan F., Bridges S.M., Peng Z. (2011). Comparison of four ChIP-Seq analytical algorithms using rice endosperm H3K27 trimethylation profiling data. PLoS ONE.

[21] Sasidharan Nair V., Saleh R., Toor S.M., Taha R.Z., Ahmed A.A., Kurer M.A., Murshed K., Alajez N.M., Abu Nada M., Elkord E. (2020). Transcriptomic profiling disclosed the role of DNA methylation and histone modifications in tumor-infiltrating myeloid-derived suppressor cell subsets in colorectal cancer. Clin. Epigenetics.

[22] Huh J.W., Lee J.H., Kim H.R. (2012). Prognostic significance of tumor-infiltrating lymphocytes for patients with colorectal cancer. Arch. Surg..

[23] Ropponen K.M., Eskelinen M.J., Lipponen P.K., Alhava E., Kosma V.M. (1997). Prognostic value of tumour-infiltrating lymphocytes (TILs) in colorectal cancer. J. Pathol..

[24] Scurr M., Gallimore A., Godkin A. (2012). T cell subsets and colorectal cancer: Discerning the good from the bad. Cell Immunol..

[25] Fountzilas E., Kotoula V., Tikas I., Manousou K., Papadopoulou K., Poulios C., Karavasilis V., Efstratiou I., Pectasides D., Papaparaskeva K. (2018). Prognostic significance of tumor genotypes and CD8+ infiltrates in stage I-III colorectal cancer. Oncotarget.

[26] Khan O., Giles J.R., McDonald S., Manne S., Ngiow S.F., Patel K.P., Werner M.T., Huang A.C., Alexander K.A., Wu J.E. (2019). TOX transcriptionally and epigenetically programs CD8(+) T cell exhaustion. Nature.

[27] Taylor E.S., McCall J.L., Girardin A., Munro F.M., Black M.A., Kemp R.A. (2016). Functional impairment of infiltrating T cells in human colorectal cancer. Oncoimmunology.

[28] Wherry E.J., Kurachi M. (2015). Molecular and cellular insights into T cell exhaustion. Nat. Rev. Immunol..

[29] Jiang P., Gu S., Pan D., Fu J., Sahu A., Hu X., Li Z., Traugh N., Bu X., Li B. (2018). Signatures of T cell dysfunction and exclusion predict cancer immunotherapy response. Nat. Med..

[30] Bonaventura P., Shekarian T., Alcazer V., Valladeau-Guilemond J., Valsesia-Wittmann S., Amigorena S., Caux C., Depil S. (2019). Cold Tumors: A Therapeutic Challenge for Immunotherapy. Front. Immunol..

[31] Thomas D.A., Massague J. (2005). TGF-beta directly targets cytotoxic T cell functions during tumor evasion of immune surveillance. Cancer Cell.

[32] Saleh R., Elkord E. (2020). FoxP3+ T regulatory cells in cancer: Prognostic biomarkers and therapeutic targets. Cancer Lett..

[33] di Bari M.G., Lutsiak M.E., Takai S., Mostbock S., Farsaci B., Semnani R.T., Wakefield L.M., Schlom J., Sabzevari H. (2009). TGF-beta modulates the functionality of tumor-infiltrating CD8+ T cells through effects on TCR signaling and Spred1 expression. Cancer Immunol. Immunother..

[34] Tauriello D.V.F., Palomo-Ponce S., Stork D., Berenguer-Llergo A., Badia-Ramentol J., Iglesias M., Sevillano M., Ibiza S., Canellas A., Hernando-Momblona X. (2018). TGFbeta drives immune evasion in genetically reconstituted colon cancer metastasis. Nature.

[35] Mariathasan S., Turley S.J., Nickles D., Castiglioni A., Yuen K., Wang Y., Kadel E.E., Koeppen H., Astarita J.L., Cubas R. (2018). TGFbeta attenuates tumour response to PD-L1 blockade by contributing to exclusion of T cells. Nature.

[36] Saleh R., Elkord E. (2020). Acquired resistance to cancer immunotherapy: Role of tumor-mediated immunosuppression. Semin. Cancer Biol..

[37] Gerstel D., Wegwitz F., Jannasch K., Ludewig P., Scheike K., Alves F., Beauchemin N., Deppert W., Wagener C., Horst A.K. (2011). CEACAM1 creates a pro-angiogenic tumor microenvironment that supports tumor vessel maturation. Oncogene.

[38] Lavergne E., Combadiere C., Iga M., Boissonnas A., Bonduelle O., Maho M., Debre P., Combadiere B. (2004). Intratumoral CC chemokine ligand 5 overexpression delays tumor growth and increases tumor cell infiltration. J. Immunol..

[39] Horvath C.M. (2004). The Jak-STAT pathway stimulated by interferon gamma. Sci. STKE.

[40] Sercan O., Stoycheva D., Hammerling G.J., Arnold B., Schuler T. (2010). IFN-gamma receptor signaling regulates memory CD8+ T cell differentiation. J. Immunol..

[41] Toyoda H., Ido M., Nakanishi K., Nakano T., Kamiya H., Matsumine A., Uchida A., Mizutani H., de Beaucoudrey L., Vogt G. (2010). Multiple cutaneous squamous cell carcinomas in a patient with interferon gamma receptor 2 (IFN gamma R2) deficiency. J. Med. Genet..

[42] Liu Y., Bao C., Wang L., Han R., Beier U.H., Akimova T., Cole P.A., Dent S.Y.R., Hancock W.W. (2019). Complementary Roles of GCN5 and PCAF in Foxp3+ T-Regulatory Cells. Cancers.

[43] Villagra A., Sotomayor E.M., Seto E. (2010). Histone deacetylases and the immunological network: Implications in cancer and inflammation. Oncogene.

[44] Xiao H., Jiao J., Wang L., O’Brien S., Newick K., Wang L.C., Falkensammer E., Liu Y., Han R., Kapoor V. (2016). HDAC5 controls the functions of Foxp3(+) T-regulatory and CD8(+) T cells. Int. J. Cancer.

[45] Ptaschinski C., Mukherjee S., Moore M.L., Albert M., Helin K., Kunkel S.L., Lukacs N.W. (2015). RSV-Induced H3K4 Demethylase KDM5B Leads to Regulation of Dendritic Cell-Derived Innate Cytokines and Exacerbates Pathogenesis In Vivo. PLoS Pathog..

[46] Li Q., Zou J., Wang M., Ding X., Chepelev I., Zhou X., Zhao W., Wei G., Cui J., Zhao K. (2014). Critical role of histone demethylase Jmjd3 in the regulation of CD4+ T-cell differentiation. Nat. Commun..

[47] De Araujo-Souza P.S., Hanschke S.C., Viola J.P. (2015). Epigenetic control of interferon-gamma expression in CD8 T cells. J. Immunol. Res..

[48] Deschoolmeester V., Baay M., Lardon F., Pauwels P., Peeters M. (2011). Immune Cells in Colorectal Cancer: Prognostic Relevance and Role of MSI. Cancer Microenviron..

[49] Ling A., Lundberg I.V., Eklof V., Wikberg M.L., Oberg A., Edin S., Palmqvist R. (2016). The infiltration, and prognostic importance, of Th1 lymphocytes vary in molecular subgroups of colorectal cancer. J. Pathol. Clin. Res..

[50] Amicarella F., Muraro M.G., Hirt C., Cremonesi E., Padovan E., Mele V., Governa V., Han J., Huber X., Droeser R.A. (2017). Dual role of tumour-infiltrating T helper 17 cells in human colorectal cancer. Gut.

[51] De Simone V., Pallone F., Monteleone G., Stolfi C. (2013). Role of TH17 cytokines in the control of colorectal cancer. Oncoimmunology.

[52] Sasidharan Nair V., Elkord E. (2018). Immune checkpoint inhibitors in cancer therapy: A focus on T-regulatory cells. Immunol. Cell Biol..

[53] Medema J.P., de Jong J., Peltenburg L.T., Verdegaal E.M., Gorter A., Bres S.A., Franken K.L., Hahne M., Albar J.P., Melief C.J. (2001). Blockade of the granzyme B/perforin pathway through overexpression of the serine protease inhibitor PI-9/SPI-6 constitutes a mechanism for immune escape by tumors. Proc. Natl. Acad. Sci. USA.

[54] Ganesh K., Stadler Z.K., Cercek A., Mendelsohn R.B., Shia J., Segal N.H., Diaz L.A. (2019). Immunotherapy in colorectal cancer: Rationale, challenges and potential. Nat. Rev. Gastroenterol. Hepatol..

[55] Parekh S., Ziegenhain C., Vieth B., Enard W., Hellmann I. (2016). The impact of amplification on differential expression analyses by RNA-seq. Sci. Rep..

