# Supplementary material for: Transcriptome of Tumor-Infiltrating T Cells in Colorectal Cancer Patients Uncovered a Unique Gene Signature in CD4+ T Cells Associated with Poor Disease-Specific Survival
Source: Vaccines (Basel). 2021 Apr 1;9(4):334. doi: 10.3390/vaccines9040334 (PMC8065799; doi:10.3390/vaccines9040334)
Supplement: Supplementary file 1 [file vaccines-09-00334-s001.zip › Supplementary Figure 1.pdf]

Supplementary material

Transcriptome of tumor-infiltrating T cells in colorectal cancer patients uncovered a unique gene signature in CD4+ T cells associated with poor disease-specific survival

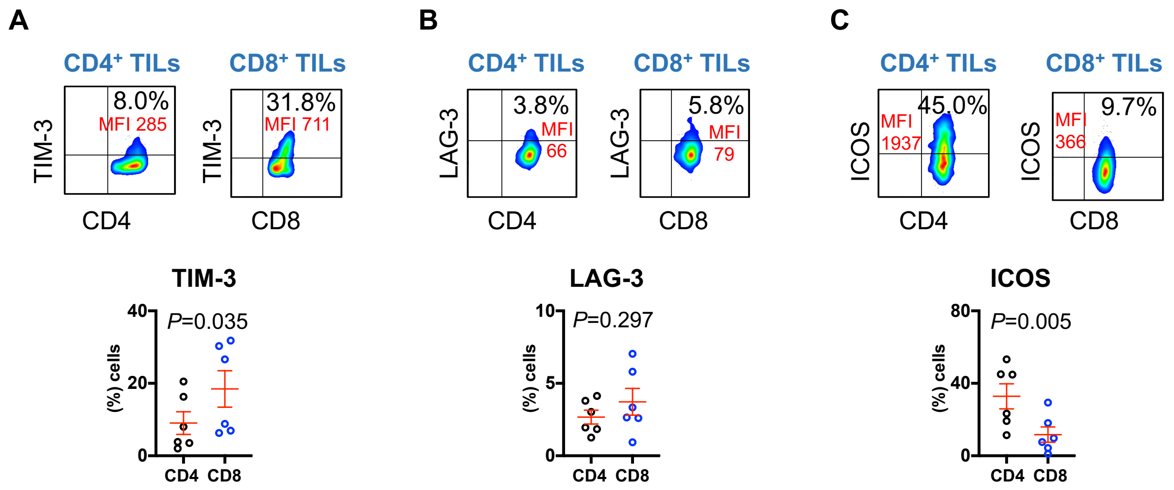

Figure S1. Expression levels of selected dysregulated genes on CD4+ and CD8+ TILs. We validated protein expression of selective immune checkpoint genes; HAVCR2 (gene for TIM-3), ICOS and LAG-3 in 6 CRC patients out of our patient cohort. Representative flow cytometric plots show the percentage and mean fluorescent intensity (MFI), and scatter plots show differences in percentage of TIM-3 **(A)**, ICOS **(B)** and LAG-3 **(C)** expressing CD4+ and CD8+ TILs from 6 CRC patients.
