# Supplementary material for: Transcriptome of Tumor-Infiltrating T Cells in Colorectal Cancer Patients Uncovered a Unique Gene Signature in CD4+ T Cells Associated with Poor Disease-Specific Survival
Source: Vaccines (Basel). 2021 Apr 1;9(4):334. doi: 10.3390/vaccines9040334 (PMC8065799; doi:10.3390/vaccines9040334)
Supplement: Supplementary file 1 [file vaccines-09-00334-s001.zip › Supplementary Table 2.docx]

**Supplementary Table 2 : CD4^+^ TILs Poor Prognosis Score (ppScore) gene list**

| **Upregulated Genes** | |  | **Downregulated genes** |
| --- | --- | --- | --- |
| *ANK1* | *SELP* |  | *GML* |
| *CPM* | *BTBD11* |  | *SLC25A15* |
| *CCR7* | *THADA* |  | *GZMB* |
| *TNFRSF25* | *ADAM19* |  | *SPNS3* |
| *PIM2* | *RGS6* |  | *BCAS4* |
| *LRRC32* | *MYOF* |  | *SLC17A9* |
| *TRIM45* | *MAGEH1* |  | *MFSD6L* |
| *PCBP3* | *INF2* |  | *PLCXD2* |
| *IFNGR2* | *TNFSF13B* |  | *SCUBE1* |
| *SLC16A10* | *NTRK3* |  | *CCRL2* |
| *FAM124B* | *MALT1* |  | *TNIP3* |
| *THEM4* | *ECSCR* |  | *SLC7A4* |
| *MAF* | *OSBPL1A* |  | *LANCL3* |
| *ADI1* | *PLK3* |  | *MYO6* |
| *GADD45A* | *S100Z* |  | *LRRC43* |
| *TMEM136* | *PBXIP1* |  | *ADAM22* |
| *CCR6* | *KLHL5* |  | *FAM83D* |
| *PLA1A* | *SELL* |  | *USP28* |
| *MCF2L2* | *FAM153A* |  | *CXXC5* |
| *HSD11B1* | *ALPK2* |  | *CMC1* |
| *CXorf21* | *SECTM1* |  |  |
| *TCN2* | *GNA15* |  |  |
| *AHSP* | *NTRK2* |  |  |
| *MSRB2* | *NETO2* |  |  |
| *LGALS12* | *CGA* |  |  |
| *SAT1* | *FAM153B* |  |  |
| *PLAT* | *CYSLTR1* |  |  |
| *ACOXL* | *MYO16* |  |  |
| *UXS1* | *KEL* |  |  |
| *PRSS1* |  |  |  |
| *CORO1B* |  |  |  |
| *TMEM173* |  |  |  |
| *FAM78B* |  |  |  |
| *ATP1A4* |  |  |  |
| *TRAF3IP2* |  |  |  |
| *CEACAM1* |  |  |  |
| *AKR1C1* |  |  |  |
| *TWIST1* |  |  |  |
| *VDR* |  |  |  |
| *TLR2* |  |  |  |
