# Supplementary material for: Transcriptome of Tumor-Infiltrating T Cells in Colorectal Cancer Patients Uncovered a Unique Gene Signature in CD4+ T Cells Associated with Poor Disease-Specific Survival
Source: Vaccines (Basel). 2021 Apr 1;9(4):334. doi: 10.3390/vaccines9040334 (PMC8065799; doi:10.3390/vaccines9040334)
Supplement: Supplementary file 1 [file vaccines-09-00334-s001.zip › Supplementary Table 3.docx]

**Supplementary Table 3 - Patient ppScores**

| **Sample** | **Stage** | **ppScore** |
| --- | --- | --- |
| Patient 1 | IV | **High ppScore** |
| Patient 2 | II |  |
| Patient 3 | IV |  |
| Patient 4 | III |  |
| Patient 5 | I |  |
| Patient 6 | III |  |
| Patient 7 | IV |  |
| Patient 8 | IV |  |
| Patient 9 | II |  |
| Patient 10 | I | **Low ppScore** |
| Patient 11 | III |  |
| Patient 12 | IV |  |
| Patient 13 | II |  |
| Patient 14 | I |  |
| Patient 15 | I |  |
| Patient 16 | II |  |
| Patient 17 | II |  |
| Patient 18 | I |  |
