# Supplementary material for: Transcriptome of Tumor-Infiltrating T Cells in Colorectal Cancer Patients Uncovered a Unique Gene Signature in CD4+ T Cells Associated with Poor Disease-Specific Survival
Source: Vaccines (Basel). 2021 Apr 1;9(4):334. doi: 10.3390/vaccines9040334 (PMC8065799; doi:10.3390/vaccines9040334)
Supplement: Supplementary file 1 [file vaccines-09-00334-s001.zip › Supplemetary Table 1.docx]

**Supplementary Table 1: T cell-related genes**

| **Gene Name** | **Log₂ fold change** | ***P* value** |
| --- | --- | --- |
| *TNFRSF4* | 5.53101558 | 0 |
| *CTLA4* | 2.19394311 | 2.3981E-14 |
| *ICOS* | 2.07001643 | 0 |
| *CD28* | 2.06381342 | 0 |
| *TNFRSF18* | 1.59909281 | 1.0438E-07 |
| *BTLA* | 1.44325432 | 2.1536E-08 |
| *CD274* | 1.37110892 | 4.2806E-07 |
| *VSIR* | 0.91145763 | 0.00077364 |
| *TIGIT* | 0.06987619 | 0.70759951 |
| *CD27* | 0.04567915 | 0.84335468 |
| *LGALS9* | -0.0711779 | 0.80532491 |
| *TNFRSF9* | -0.1745213 | 0.53066396 |
| *PDCD1* | -0.3925363 | 0.23976114 |
| *TNFSF14* | -0.6277746 | 0.01830853 |
| *TMIGD2* | -0.7614742 | 0.05602474 |
| *HAVCR2* | -1.2251415 | 7.9291E-06 |
| *LAG3* | -1.8127297 | 0 |
| *KIR2DS4* | -5.5675924 | 0 |
| *CD4* | 8.1096467 | 0 |
| *CCR8* | 6.60192003 | 0 |
| *LRRC32* | 5.48364546 | 2.2204E-16 |
| *FOXP3* | 4.70249594 | 0 |
| *CD40LG* | 4.30673788 | 0 |
| *IL2RA* | 3.6293085 | 0 |
| *CCR6* | 2.54158271 | 1.0436E-14 |
| *CTLA4* | 2.19394311 | 2.3981E-14 |
| *ICOS* | 2.07001643 | 0 |
| *IL7R* | 1.03517695 | 0.00038557 |
| *TNFRSF1B* | 0.59902663 | 0.00161616 |
| *ITGA4* | 0.32375753 | 0.10702324 |
| *IKZF2* | 0.3223726 | 0.16694881 |
| *STAT5A* | 0.32001961 | 0.0482982 |
| *STAT5B* | 0.3170612 | 0.02866492 |
| *TGFB1* | -0.1393947 | 0.6033956 |
| *PTPRC* | -0.1460176 | 0.44409799 |
| *HLA-DRA* | -1.342042 | 4.9382E-06 |
| **Gene Name** | **Log₂ fold change** | **P-value** |
| *LAG3* | -1.8127297 | 0 |
| *ITGAE* | -1.9072772 | 1.4322E-14 |
| *TWIST1* | 4.68528352 | 9.6867E-13 |
| *MMP9* | 2.82087537 | 5.691E-13 |
| *TCF4* | 1.80299164 | 5.6334E-07 |
| *CDH1* | 1.30672654 | 0.00203757 |
| *ZEB1* | 0.15305031 | 0.31982439 |
| *SNAI2* | -0.238433 | 0.68682683 |
| *ZEB2* | -1.0480196 | 0.00017982 |
| *VEGFA* | -1.4317951 | 0.00395745 |
| *IFNG* | -0.8433668 | 0.01841801 |
| *TBX21* | -0.9032358 | 0.00047062 |
| *IRF8* | -1.3103064 | 0.00051586 |
| *EOMES* | -2.8660478 | 0 |
| *MAF* | 1.78869961 | 1.2212E-15 |
| *GATA3* | 0.67240504 | 0.00019966 |
| *IRF4* | 0.6623956 | 0.00681258 |
| *IKZF1* | 0.32554113 | 0.07057972 |
| *RORC* | 1.4741781 | 1.1465E-05 |
| *RORA* | 0.9184764 | 1.2822E-05 |
| *HIF1A* | 0.51580926 | 0.00548668 |
| *TOX* | -0.552466 | 0.00893168 |
| *TOX3* | -2.1905368 | 7.6351E-05 |
| *PDCD1* | -0.3925363 | 0.23976114 |
| *HAVCR2* | -1.2251415 | 7.9291E-06 |
| *LAG3* | -1.8127297 | 0 |
| *KLRG1* | -1.7036113 | 7.6394E-13 |
| *NCAM1* | -1.2522401 | 0.00065591 |
| *IL1R2* | 6.5659066 | 0 |
| *IL1R1* | 6.1578736 | 0 |
| *IL6R* | 5.3865331 | 0 |
| *IL22* | 5.33262778 | 0 |
| *IL1RL1* | 4.65662233 | 0 |
| *IL21* | 4.39698102 | 0 |
| *IL17F* | 4.23849917 | 2.2204E-16 |
| *IL17RB* | 3.81583622 | 0 |
| *IL2RA* | 3.6293085 | 0 |
| *IL17A* | 2.57973746 | 1.5353E-06 |
| **Gene Name** | **Log₂ fold change** | **P-value** |
| *IL31* | 2.10517632 | 0.01671896 |
| *IL26* | 2.0154683 | 2.2064E-06 |
| *IL4* | 1.98266068 | 0.00125987 |
| *IL23R* | 1.97086243 | 2.3841E-10 |
| *IL7* | 1.94233674 | 1.0103E-08 |
| *IL23A* | 1.77206998 | 3.3181E-08 |
| *IL3RA* | 1.74536143 | 2.4492E-07 |
| *IL2* | 1.60886962 | 3.1849E-07 |
| *IL5* | 1.38557027 | 0.04902992 |
| *IL18R1* | 1.31510367 | 6.9254E-10 |
| *IL10* | 1.20974896 | 0.0031007 |
| *IL24* | 0.78309242 | 0.00578232 |
| *IL9R* | -1.9662844 | 2.4618E-12 |
